# MiRNAs as Regulators of Immune Cells in the Tumor Microenvironment of Ovarian Cancer

**DOI:** 10.3390/cells13161343

**Published:** 2024-08-13

**Authors:** Miłosz Wilczyński, Jacek Wilczyński, Marek Nowak

**Affiliations:** 1 Department of Operative Gynecology, Endoscopy and Gynecologic Oncology, Polish Mother’s Health Center-Research Institute, 281/289 Rzgowska Str., 93-338 Lodz, Poland; 2 Department of Gynecological Surgery and Gynecological Oncology, Medical University of Lodz, 4 Kosciuszki Str., 90-419 Lodz, Poland; jacek.wilczynski@umed.lodz.pl; 3 Department of Operative Gynecology and Gynecologic Oncology, Polish Mother’s Health Center-Research Institute, 281/289 Rzgowska Str., 93-338 Lodz, Poland; marek.nowak@iczmp.edu.pl

**Keywords:** tumor microenvironment, ovarian cancer, miRNAs

## Abstract

Ovarian cancer is one of the leading causes of cancer deaths among women. There is an ongoing need to develop new biomarkers and targeted therapies to improve patient outcomes. One of the most critical research areas in ovarian cancer is identifying tumor microenvironment (TME) functions. TME consists of tumor-infiltrating immune cells, matrix, endothelial cells, pericytes, fibroblasts, and other stromal cells. Tumor invasion and growth depend on the multifactorial crosstalk between tumor cells and immune cells belonging to the TME. MiRNAs, which belong to non-coding RNAs that post-transcriptionally control the expression of target genes, regulate immune responses within the TME, shaping the landscape of the intrinsic environment of tumor cells. Aberrant expression of miRNAs may lead to the pathological dysfunction of signaling pathways or cancer cell-regulatory factors. Cell-to-cell communication between infiltrating immune cells and the tumor may depend on exosomes containing multiple miRNAs. MiRNAs may exert both immunosuppressive and immunoreactive responses, which may cause cancer cell elimination or survival. In this review, we highlighted recent advances in the field of miRNAs shaping the landscape of immune cells in the TME.

## 1. Introduction

Ovarian cancer (OC) is the 8th most common cancer in women worldwide. There were more than 314,000 new cases of OC in 2020 [1]. Due to the widespread use of oral contraceptives, the incidence rate of OC has slightly declined in many populations. However, countries with transitioning economies will experience a significant rise in OC cases in upcoming years [1]. OC is one of the leading causes of cancer deaths among women. Most OC cases manifest an advanced stage (III and IV stage according to FIGO), which is the main reason for unfavorable prognosis among OC patients. The majority of ovarian malignancies are derived from epithelial cells, and high-grade tumors cause more than 70% of these cases. Despite the surgical advances in the field of cytoreductive surgery, together with the implementation of platinum/taxane-based chemotherapy, the recurrence rate and resistance to standard chemotherapy regimens are still the most troublesome issues in OC patients. Oxidative stress is a constant feature of the cancer microenvironment. Chemoresistance of OC partially depends on ovarian cancer cells’ increased antioxidant capacity. Thus, OC cells protect themselves by developing antioxidant responses, such as upregulating Solute Carrier Family 7 Member 11 (SLC7A11), leading to changes in cysteine import to cancer cells and maintaining redox homeostasis [2]. Recent molecular advances have led to a significant improvement in outcomes. Implementation of poly (ADP-ribose) polymerase (PARP) inhibitors (PARPi) has proven to be an effective strategy in maintenance therapy, leading to better long-term prognosis, especially among OC patients with a homologous recombination deficit [3]. The example of PARPi has shown us that the clinical utilization of diverse molecular/pathological mechanisms associated with tumor progression or survival may benefit patients. Therefore, there is still a significant need to use recent advancements in tumor biology.

Long ago, we thought a tumor was only a group of pathological cells presenting proliferative and metastatic features. Now we know that tumors, especially OC, are a heterogeneous collection of tumor cells, infiltrated by multiple immune or stromal cells and numerous secreted factors and an extracellular matrix. The tumor microenvironment (TME) is a dynamic and constantly evolving entity that creates hospitable conditions in host tissues to support tumor growth, cancer progression, and the ability to form metastases. TME consists of numerous composites, such as immune cells, stromal cells, extracellular matrix, exosomes, and neovascular blood vessels. It seems that TME is not only a derivative of the tumor but a promoter of cancer progression itself [4,5]. There are several mechanisms in which immunosuppression may be introduced in the tumors, such as CD8^+^ T cells inhibition by regulatory T cells (Tregs), secretion of IL-10/IL-6 with inhibition of PD-1 receptor, immunosuppressive actions of myeloid-derived suppressor cells (MDSCs), tumor-associated macrophages (TAMs), or cancer-associated fibroblasts (CAFS). The orchestra of TME compounds may be altered in several ways, including the expression of miRNAs [6].

MiRNAs are small, single-stranded, non-coding RNAs, typically 18–25 long nucleotides, conserved across species through evolution. Mature miRNAs are integrated into the RNA-induced silencing complex (RISC), where they bind to the 3′ untranslated regions (UTRs) of their target mRNAs [7,8]. This interaction leads to the posttranscriptional repression or activation of translation or even mRNA degradation. MiRNAs can modulate gene expression and are involved in various cellular processes, including proliferation, differentiation, epithelial-to-mesenchymal transition (EMT), and apoptosis [8]. There is substantial evidence indicating that miRNAs are critical in the processes of carcinogenesis, tumor development, and metastasis. Numerous studies have documented that miRNAs are dysregulated in various cancers. The abnormal expression of miRNAs has been observed in several types of tumors and is often linked to disease stage and clinical outcomes [9].

MiRNAs also play a pivotal role in regulating TME, leading to tumor angiogenesis, shaping tumor immunogenicity, and interstitial interactions [10,11,12]. Immune cells are critical components of TME, taking part both in the suppression of cancer cells and the promotion of tumor expansion. They belong to adaptive and innate immune systems. The immune tumor environment comprises T cells (CD4^+^, CD8^+^, Tregs), natural killer cells (NK cells), macrophages (TAMs—tumor-associated macrophages), dendritic cells, and MDSCs (myeloid-derived suppressor cells). Other than the immune system compounds, TME is created by extracellular matrix and stromal cells, such as fibroblasts (CAFs—cancer-associated fibroblasts) or endothelial cells. The stromal part of TME is also responsible for the secretion of many factors that influence angiogenesis, proliferation, invasion, and metastasis. However, in this review, we focus on those miRNAs that regulate only immune cells infiltrating the tumor and thus forming the immune landscape of TME, with particular emphasis on ovarian cancer.

## 2. MiRNAs as Modulators of Immune Response in the TME: General Remarks and Examples from Studies outside the Ovarian Cancer Spectrum

**Figure 1:**
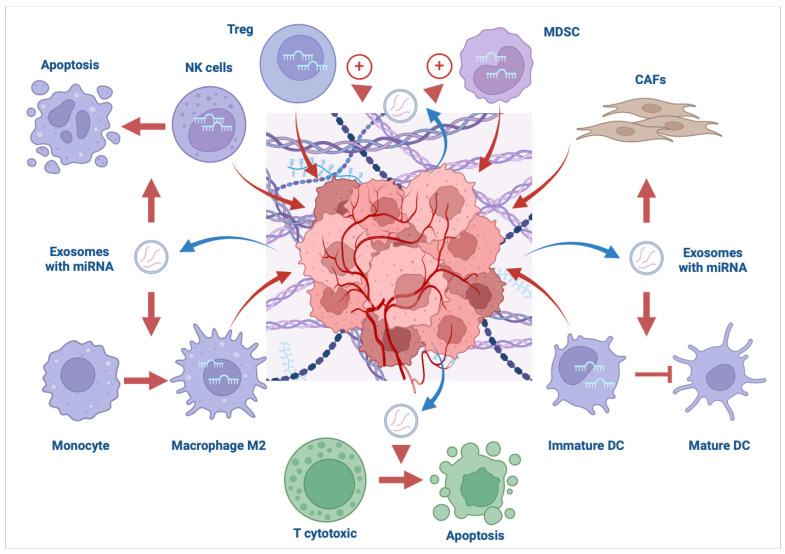
miRNAs regulate both TME and tumor, being part of a multilayered and mutual network between cancer and immune cells. Tumor-derived exosomes containing miRNAs modulate the actions of immune cells in the TME. Up- or downregulation of certain miRNAs in immune cells being part of the TME also affects tumor growth and progression. Such phenomena may cause immunotolerance or tumor elimination. Immune cells: natural killer cells—NK cells; myeloid-derived suppressor cells—MDSC; regulatory T cells—Treg; CD8^+^ T cells—T cytotoxic; dendritic cells—DC; cancer-associated fibroblasts—CAFs.

MiRNAs serve as modulators of TME, taking part in multifaced crosstalk between immune cells and tumors. They can both exert immunosuppressive and antitumorigenic actions, leading to cancer growth and progression or its elimination. Moreover, tumor-derived miRNAs might target immune cells, directly influencing their immune responses. Data on miRNAs’ actions in TME are derived from diverse studies with different types of tumors or based on both in vitro and in vivo studies. Below, we present examples of the most studied miRNAs in TME, which indicates their pivotal role in regulating immune cells within TME. Mechanisms by which miRNAs mediate the regulation of TME are multilayered and involve all kinds of immune cells infiltrating the tumor cells [13,14,15,16]. Schematic miRNA-dependent regulation of the TME and tumor is presented in Figure 1.

### 2.1. T Cells

There are several subpopulations of T cells in humans. Th2 and Th17 cells are not directly involved in tumor immunity, taking part in immune responses against fungi, parasites, or bacteria. CD8^+^ T cells detect abnormal tumor antigens responsible for cancer cells’ cytotoxic destruction. Moreover, CD8^+^ cells produce interferon-gamma, causing suppression of angiogenesis. Abundant infiltration of tumors by CD8^+^ T cells is a sign of a favorable prognosis [17]. CD4^+^ Th1 cells play an important role in antitumor immunoactivity, supporting cytotoxic CD8^+^ cells by secreting interleukin-2 (IL-2) and interferon-gamma. Regulatory T cells (Tregs) are the basis of processes associated with immunosuppression. In TME, Tregs promote tumor growth and progression by dampening antitumor activity [5].

Expression of miRNA-155 and miRNA-146 is upregulated by proinflammatory stimuli such as interleukin-1 (IL-1), tumor necrosis factor-alpha (TNFalpha), and Toll-like receptors (TLRs) [18]. MiRNAs may also interfere with interferon-gamma signaling, as miRNA-155 contributes to Th1 differentiation by targeting interferon-gamma α-chain [19]. Moreover, miRNA-155 and miRNA-26 may play an essential role in the antitumor effect of CD8^+^ T cells. Above-mentioned miRNAs may regulate CD8^+^ T cell secretion of interferon-gamma, which is the key to antitumor responses in TME [20,21]. Huffaker et al. showed that miRNA-155–deficient mice, after administration of syngeneic EL4-luc lymphoma cells subcutaneously, were characterized by more considerable tumor growth compared to Wt mice. Authors reported that in the spleens and lymph nodes of mice with EL4-luc tumors, a lack of miR-155 led to a significant reduction of interferon-gamma-producing CD4^+^ T cells compared to Wt controls. Regarding CD8^+^ T cells, Huffaker et al. examined interferon-gamma expression by CD8^+^ T cells following their activation in vitro and observed defective interferon-gamma mRNA levels in the absence of miRNA-155 [21]. These data show that miRNA-155 significantly impacts interferon-gamma-dependent antitumor immunity within CD4^+^ and CD8^+^ T cells.

Another example of miRNAs’ functions in the TME is the miRNA-17-92 cluster, whose deficiency in CD4^+^ T cells leads to impaired response of Th1 cells towards B16 melanoma tumor cells and turns off CD4^+^ T cells cooperation with CD8^+^ T cells. MiRNA-19b and miRNA-17, both members of the miR-17-92 cluster, play a pivotal role in inhibiting tumor growth. MiRNA-19b targets phosphatase and tensin homolog deleted on chromosome ten (PTEN), promotes the proliferation of Th1 cells, induces interferon-gamma secretion, and impairs the differentiation of iTreg. MiRNA-17 targets cAMP-responsive element binding protein (CREB)1 and inhibits the differentiation of iTreg [22]. These processes contribute to strengthening antitumor immunoactivity.

MiRNAs may also influence the Treg cells’ functions. Qin et al. reported that miRNA-126 silencing reduced the expression of Foxp3 on Tregs, which was accompanied by decreased production of IL-10 and TGF-β, leading to impairment of Tregs’ suppressive capabilities. Mechanistic evidence showed that miRNA-126 targets p85β, which may cause alteration of the phosphoinositide 3-kinase (PI3K)/protein kinase B (Akt) pathway and thus impair Tregs’ induction [23].

Tumor-derived exosomal miRNAs may also shape the immune system of TME. Cancer cells conduct suppressive actions through cancer cell-secreted exosomes containing several miRNAs. Treg cells, recipients of miRNA-214, present reduced PTEN levels and secrete higher amounts of IL-10, which contributes to the promotion of tumor growth [24].

Such miRNAs as miRNA-139 or miRNA-342 are involved in regulating perforin expression. Perforin lytic activity is one of the critical mechanisms of CD8^+^ T cells’ actions toward cancer cells [25].

### 2.2. Macrophages

Macrophages are critical innate immune system compounds responsible for pathogen phagocytosis and antigen presentation. According to their ability to induce or suppress inflammatory processes, macrophages might be M1 or M2 macrophages, respectively. TAMs abundantly infiltrate the TME, being both anti- and pro-tumoral. Over time, M2-polarized macrophages become more present in the TME, exerting immuno-suppressive features. Evidence shows that M1/M2 polarization may depend on miRNAs’ expression levels. Upregulation of miRNA-155 in TAMs leads to a shift from suppressive M2 macrophages into anti-tumoral type of M1 macrophages [26]. Moreover, M1/M2 polarization may occur upon the influence of miRNA-19a-3p, which targets FRA-1 (Fos-related antigen1) and contributes to the proinflammatory M1 switch [27].

Another interesting phenomenon connected to macrophages’ actions within TME is miRNAs’ exosome transfer between TAMS and cancer cells. MiRNa-let-7b transfer in microvesicles from HCC (hepatocellular carcinoma) cells to macrophages results in the downregulation of IL-6 [28]. Tumor-secreted miRNA-29 and miRNA-21 may change TME responses in macrophages through TLR-8 signaling [29]. Furthermore, miRNA-451 and miRNA-21 in extracellular vesicles released from primary human glioblastoma (GBM) to macrophages decreased the transcription factor c-Myc [30]. According to Huang et al., miRNA-146a can promote M2 polarization by targeting neurogenic locus notch homolog protein 1 (NOTCH1) [31].

Wang et al. demonstrated that M2 macrophage-derived exosomes exhibited a high expression level of miRNA-3679-5p in lung cancer cells. MiRNA-3679-5p downregulated neural precursor cell expressed developmentally downregulated gene 4-like (NEDD4L), one of the critical regulators of the c-Myc stability [32].

### 2.3. Dendritic Cells

Dendritic cells (DCs) serve as antigen-presenting cells that recognize and present antigens to T cells, initiating antigen-specific T cell activity. Within TME, dendritic cells may exert antitumorigenic responses. However, they can also participate in immunosuppressive actions leading to tumor progression. Tumors are the source of multiple soluble factors (i.e., cytokines) influencing TIDCs’ (tumor-infiltrating DCs) intracellular signaling pathways, disrupting their regular function. Liang et al. reported this example, which showed that miRNA-22 inhibits p38 protein by directly binding to its mRNA’s 3′ untranslated region (3′UTR). The p38 protein is integral to mitogen-activated protein kinases (MAPK) signaling and exerts IL-6 expression. Therefore, miRNA-22 regulates interleukin-6 (IL-6) expression through the p38 protein and MAPK intracellular signaling pathway [33]. It has been shown that miRNA-155 targets multiple immune cells within the TME. In the case of TICDs, it was reported that silencing of c-Fos expression by miRNA-155 is a conserved process required for DC maturation and function [34]. Tumor-derived exosomes may play an essential role in activating or suppressing several immune cells in TME; TIDCs are no exception. Zhou et al. investigated the influence of pancreatic cancer-derived exosomes on TLR4 and downstream cytokines via mmiRNA-203. Their results showed that TLR4, TNF-α, and interleukin-12 (IL-12) decreased under the treatment of exosomes and miRNA-203 mimics [35].

### 2.4. NK Cells

NK cells are present mainly in the bloodstream. NK cells might be provisionally divided into two subpopulations: those that secrete inflammatory cytokines and the second class that directly takes part in killing tumor cells. Similarly to other immune cells in the TME, NK cells’ function is influenced by miRNA-155. Data from lymphoma-bearing mice showed that miRNA-155 targets Src homology 2 (SH2) domain containing inositol polyphosphate 5-phosphatase 1 (SHIP1) and promotes interferon-gamma production, exerting upregulation of antitumorigenic processes [36]. Several authors reported the role of miRNAs in regulating granzyme B and perforin, proteases crucial to NK cells. It was reported that miRNA-223, miRNA27a-5p, miRNA-150, and miRNA-378 may negatively affect the antitumor actions of NK cells through perforin or granzyme regulation [37,38,39]. MiRNAs also potentially play an important role in other NK cells’ effector responses. Donatelli et al. discovered that transforming growth factor β (TGF-β)-–treated human NK cells exhibit reduced tumor cytolysis and abrogated perforin polarization to the immune synapse, losing surface expression of activating killer Ig-like receptor 2DS4 and NKp44. Mechanistic analysis indicated that TGF-β induced miRNA-183 to negatively regulate DNAX activating protein 12 kDa (DAP12), which is needed for surface NK receptor stabilization [40].

In the case of NK cells, it was also confirmed that tumor-derived miRNAs located in microvesicles are mediators between tumor and immune cells. MiRNA-23a in hypoxic microvesicles is an immunosuppressive factor targeting interferon-gamma and CD107a in NK cells [41].

### 2.5. MDSCs

Myeloid-derived suppressor cells (MDSCs) are immature cells of myeloid origin that suppress immune responses during cancer progression. MDSCs’ immunosuppressive actions occur via Tregs-mediated pathways and direct inhibition of immune cells.

One of the critical factors of MDSCs regulation is miRNA-494, which targets PTEN and activates the AKT signaling pathway. Liu et al. reported that due to tumor-derived TGF-β1 secretion, the upregulation of miRNA-494 took place. Expression of miRNA-494 was associated with enhanced CXCR4-mediated MDSCs’ chemotaxis, infiltration of MDSCs in tumor tissues, and altered PTEN-dependent apoptotic/survival signal [42]. Huang et al. reported that miRNA-34a contributes to apoptosis inhibition of MDSCs cells, potentially by targeting such genes as p2rx7, Tia1, and plekhf1 [43]. Moreover, another group demonstrated that miR-34a inhibited apoptosis through the reduced translation of N-myc without affecting the proliferation [44].

Guo et al. used reactive oxygen species (ROS), arginase activity, nitric oxide (NO), T-cell proliferation, and immunosuppressive cytokine (IL-10 and TGF-β, ELISA) levels to assess MDSCs’ expansion. The hypoxia-stimulated glioma-derived exosomes containing miRNA-10/miRNA-21 activated MDSCs by targeting RAR-related orphan receptor alpha (RORA) and PTEN [45]. Another example of miRNAs influencing the TME came from the study of Mei et al. Authors discovered that induction of miRNA-200c is possible by secretion of tumor-derived granulocyte-macrophage colony-stimulating factor (GM-CSF), and miR-200c, in turn, promotes the expansion and immune suppressive activity of MDSCs via targeting PTEN and friend of Gata 2 (FOG2), which can lead to signal transducer and activator of transcription 3 (STAT3) and PI3K/Akt activation [46].

## 3. MiRNAs as Modulators of Immune Response in the TME of Ovarian Cancer

Together with tumor cells and other compounds of the TME, immune cells form a complicated, multilayered crosstalk that leads to immunosuppression and, eventually, tumor growth. As presented above, miRNAs play a pivotal role in shaping the TME, changing immune cells’ responses, influencing signaling pathways, and participating in cell-to-cell communication through exosomes. The state of knowledge concerning the role of miRNA in immune cells infiltrating the TME needs to be improved. This section presents evidence of miRNAs’ activity within TME regarding immune cells (Table 1 and Table 2).

Exosomes (EVs) are small membrane-derived vesicles about 30–140 nm in diameter. Both tumor cells and tumor-infiltrating cells secrete exosomes, which modulate cell-to-cell communication by donating membrane receptors, specific proteins, and nucleic acids, including miRNAs.

TAMs in TME are associated with poor disease outcomes, as they exert an immunosuppressive response and allow for tumor growth and metastasis. Parayath et al. reported that hyaluronic acid-based nanoparticles encapsulating miRNA-125b (HA-PEI-miR-125b) can specifically target TAMs in the peritoneal cavity of a syngeneic ID8-VEGF ovarian cancer mouse model and can repolarize macrophages to an immune-activating phenotype [47].

Ying et al. demonstrated that epithelial ovarian cancer-derived exosomes activated macrophages to a TAM-like phenotype with Suppressor of Cytokine Signaling 3 (SOCS3)/STAT3 pathway involvement. Upregulation of miRNA-222-3p resulted in polarization of the M2 macrophage phenotype [48]. Macrophages-derived exosomes may also play an essential role in tumor immunosurveillance and drug resistance. It was reported that hypoxic epithelial ovarian cancer (EOC) cells induced macrophages into a TAM-like phenotype. Hypoxic macrophages were, in turn, a source of miRNA-223-rich exosomes, promoting ovarian cancer cells’ drug resistance via the PTEN-PI3K/AKT pathway. Moreover, it was noticed that patients with high hypoxia-inducible factor 1-alpha (HIF-1a) expression were characterized by higher CD163^+^ cell infiltration and elevated expression levels of miRNA-223 in tumors [49].

There have been published studies that showed the ability of specific miRNAs to interfere with M2 macrophages. For instance, miRNA-200b is abundantly present in exosomes detected in EOC patients’ plasma and functions as an oncogene promoting M2 polarization. Xiong et al. demonstrated that miR-200b-overexpressed macrophages-conditioned medium enhanced ovarian cancer cells’ cell viability and invasive properties. Authors concluded that miRNA-200b inhibited Kruppel-like factor 6 (KLF6), eventually decreasing the M1 polarization and causing immunosuppression, leading to the malignant phenotype of tumor cells [50].

Chen et al. found that hypoxia induces high expression of miRNA-940 in ascites from EOC patients. Authors reported that miRNA-940 in exosomes caused M2-polarization, which plays a vital role in the progression of EOC [51]. Similar results were published in another study by Chen et al. Authors revealed that EOC-derived exosomes containing miRNA-21-3p, miRNA-125b-5p, and miRNA-181d-5p were also capable of inducing the polarization of M2 macrophages, which in turn exerted immunosuppressive responses leading to EOC promotion and proliferation [52]. The role of extracellular vesicle-packaged miRNA-181c-5p from human EOC cells SKOV3 was investigated by Yang et al. SKOV3 cell-derived exosomes induced by hypoxia showed high expression of miRNA-181c-5p and promoted M2 polarization. MiRNA-181c-5p targeted Lysine acetyltransferase 2B (KAT2B), upregulated Homeobox A10 (HOXA10), and activated the JAK1/STAT3 pathway. Such actions resulted in the promotion of TAMs into the M2 phenotype. In a mouse model, miRNA-181c-5p exosomes exerted growth and metastasis of EOC cells [53]. Wang et al. demonstrated the role of miRNA-532-3p/SREBF1/PI3Kα/AKT axis. EV-packaged circATP2B4 in EOC can be transmitted to macrophages and induce M2 polarization. CircATP2B4 serves as a competing endogenous RNA of miR-532-3p to relieve the repressive effect of miR-532-3p on its target SREBF1 [54].

The oncogenic role of miRNA-1246 was investigated in several ovarian cancer cell lines and OC animal models by Kanlikilicer et al. Based on TCGA data, high miRNA-1246 and low Cav1 expression (direct target of miRNA-1246) were associated with worse overall outcomes. Authors demonstrated that when OC cells are co-cultured with macrophages, they can transfer their oncogenic miR-1246 to M2-type macrophages, leading to tumor progression [55]. M2-polarized macrophages dominate the peritoneal immune environment during EOC development. Li et al. reported that exosomes derived from M2 macrophages contained miRNA-221-3p, which, through targeting cyclin-dependent kinase inhibitor 1B (CDKN1B), contributed to EOC proliferation and G1/S transition [56].

The Treg/Th17 ratio is associated with histologic grade and may be considered a prognostic factor for the overall survival of EOC patients. Results of the study by Zhou et al. suggest that TAM-derived exosomes contain miRNA-29a-3p and miRNA-21-5p. Upon transfection of CD4^+^ T cells with equivalent miRNA mimics, it was shown that both miRNAs can suppress STAT3 and regulate Treg/Th17 cell ratio. Such discovery emphasizes the role of miRNAs in intercellular communication between TAMs and T cells. Exosomal miRNA-29a-3p and miRNA-21-5p create conditions favorable for immunosuppression and EOC progression [57]. Moreover, Lu et al. tried to investigate the role of TAM-derived exosomal miRNA-29a-3p using both in vivo experiments and xenograft models. Authors observed that inhibition of TAM-derived exosomal miRNA-29a-3p decreased PD-L1 (programmed death-ligand 1) to inhibit OC progression through the FOXO3-AKT/GSK3β pathway both in vitro and in vivo [58].

Hu et al. demonstrated that exosomes derived from TWEAK-stimulated macrophages could inhibit malignant potential EOC cells. TWEAK increased miRNA-7 expression levels in macrophages and their secreted exosomes, resulting in elevated levels of miRNA-7 in EOC cells, which reduced the EGFR/AKT/ERK1/2 pathway activity [59].

Recently, Wilkinson et al. published interesting data on miRNA-let-7i, proving that this miRNA’s utilization in overcoming tumor immunosuppression might be a promising strategy. Authors using the LinkedOmics platform for analysis of 602 human high-grade EOC tumors identified the critical role of miRNA-let7a in crosstalk between cancer cells and infiltrating immune cells. Mice models were used to assess miRNA-let7i functions in the TME. Enforced miRNA-let-7i expression significantly decreased tumor burden, increased T cell numbers in tumors, and increased CD86 expression in antigen-presenting cells (APCs) in lymph nodes. Overexpression of miRNA-let-7i might lead to an increase in the presence of T cells within tumors, an upregulation of APC activity in lymph nodes, and, thus, the promotion of antitumorigenic immune responses [60]. Other miRNAs have also been reported to influence APC cells in ovarian cancer. MiRNA-22 targets YWHAZ and blocks PI3K/Akt and MAPK signaling pathways, and miRNA-503 downregulates Bcl2 expression. Upregulation of both miRNAs reduced DCs’ survival by causing apoptosis [61]. Another evidence of miRNAs’ influence on the functions of DCs came from the study of Cubillos-Ruiz et al. In their research, the authors concentrated more on using bulged RNA that mimics the structure of endogenous miRNAs. They chose miRNA-155 mimetics, which were delivered to ovarian cancer DCs. MiRNA-155 delivery to tumor-associated DCs induced abrogation of tumor progression in established ovarian cancer mice models [62].

MiRNAs are critical in regulating other immunoreactive cells in TME, such as NKs or both CD4^+^ and CD8^+^ T cells. Xie et al. demonstrated that miRNA-20a directly binds to the 3′-untranslated region of MICA/B mRNA, resulting in its degradation. MICA/B proteins are ligands of the natural killer group 2 member D (NKG2D) receptor, which is crucial for the immune-mediated elimination of tumor cells. Their study showed that increased expression levels might suppress NK cells’ cytotoxicity. Moreover, miRNA-20a serum levels were correlated with ovarian cancer stage [63]. T cell differentiation to Th1 in TME is vital to exert immune responses towards repression of tumor growth. It was demonstrated in the murine ovarian cancer model that artesunate promoted miRNA-142 expression in peripheral CD4^+^ T cells and Th1 differentiation from CD4^+^ T cells. Artesunate upregulated miRNA-142, which suppressed Sirtuin 1 (Sirt1) levels and eventually promoted Th1 differentiation—such actions led to ovarian cancer cell apoptosis [64]. TME is a lactate-rich environment that supports Treg cells. It was demonstrated that miRNA-124 directly targets monocarboxylate transporter 1 (MCT1), essential in the metabolism of tumor-infiltrating Treg cells and reduces lactate uptake. Such actions are associated with impairment of immune-suppressive responses of Treg cells [65]. It was reported that miRNA-146a may have the ability to overcome immune suppression by enhancing CD8^+^ T cell infiltration in tumors. Chen et al. showed that miRNA-146a targets IL-1 receptor-associated kinase 1 (IRAK1) and tumor necrosis factor receptor-associated factor 6 (TRAF6) leading to NF-κB signaling inhibition. Such a phenomenon may lead to a decrease in immune suppressive neutrophil rates by negatively affecting the production of the downstream neutrophil chemoattractant, C-X-C motif chemokine ligand 1 [66]. MDSCs are crucial for repressing immune response in tumor promotion and metastasis. Zheng et al. isolated MDSCs from ovarian cancer-bearing mice and observed that the upregulation of miRNA-211, targeting C/EBP homologous protein (CHOP), resulted in the inhibition of MDCSs’ immunosuppressive actions. Moreover, overexpression of miRNA-211 was associated with an increased number of co-incubated CD4^+^ and CD8^+^ T cells [67].

Programmed death 1 protein (PD 1) is present in activated T, NK, and B cells. Interaction between PD-1 and its ligand (PD-L1) facilitates negative regulation of effector immune cells by inducing the death of cytotoxic lymphocytes in the tumor microenvironment and enhancing regulatory T-lymphocyte activity. PD-L1 is overexpressed on tumor cells, which enables the inhibition of anticancer T-cell responses. PD-1 and PD-L1 are pivotal immune checkpoints that regulate tumor growth and invasion. PD-1/PD-L1 inhibition is beneficial and might be used in immunotherapy. MiRNAs related to the TME can influence PD-1/PD-L1 interactions and interfere with immunosuppressive responses against the cancer cells. Xu et al. investigated the role of miRNA-424(322) in regulating the PD-L1/PD-1 and CD80/CTLA-4 pathways in chemoresistant ovarian cancer. Authors reported that high levels of miR-424(322) in the tumors were positively correlated with progression-free survival. Mechanistic investigations revealed that miRNA-424(322) inhibited PD-L1 and CD80 expression. Restoration of miRNA-424(322) expression reversed chemoresistance through PD-L1 blockage [68].

The role of miRNA-576-3p in cisplatin sensitivity of ovarian cancer cells was reported by Zuo et al. In vitro studies using cisplatin-resistant ovarian cancer cell lines showed that miRNA-576-3p targets PD-L1 and cyclin D1. Their results proved that upregulation of miRNA-576-3p can increase cisplatin chemosensitivity of ovarian cancer cells by inhibiting PD-L1 and cyclin D1 [69]. The miRNA-200 family is considered a significant regulator of migration and invasion of EOC cells by promoting mesenchymal-epithelial transition. However, miRNA-200c-3p may also target the immune checkpoint PD-L1. Anastasiadou et al. assessed the effect of miRNA-200c-3p target genes in miRNA-200c transfected SKOV3 cells untreated and treated with olaparib and ionizing radiation alone. Results demonstrated that miRNA-200c-3p reduced PD-L1, c-Myc, and β-catenin expression levels, which sensitized ovarian cancer cells to both types of treatment and was associated with less tumorigenic microenvironment [70]. Xi et al. identified PD-L1 as a functional target of miRNA-155-5p. Authors discovered a novel mechanism in which high levels of ROS (reactive oxygen species) downregulated exosomal miRNA-155-5p and, thus, influenced the regulation of PD-L1. Delivery of tumor exo-miR-155-5p in immune-intact mice suppressed ovarian cancer progression and macrophage infiltration and activated CD8^+^ T cell function. Lower intake of exosomal miR-155-5p by macrophages created an immunosuppressive microenvironment characterized by upregulation of PD-L1 [71]. Another example of an association between miRNAs and PD-L1 regulation is a study by Zuo et al. The authors described a miRNA-34a-5p/PD-L1 axis, which regulates cisplatin resistance of ovarian cancer cells. Their analysis demonstrated that miR-34a-5p negatively regulated the expression of PD-L1 by targeting its 3′-untranslated region [72]. Feng et al. studied the role of miRNA-92 in ovarian cancer. They found that miRNA-92 is overexpressed in ovarian cancer tissue compared with normal cancer tissue.

Moreover, the authors identified the large tumor suppressor kinase 2 (LATS2) gene as a target of miRNA-92. Downregulation of LATS via miRNA-92 resulted in increased translocation of Yes1 associated transcriptional regulator (YAP1) and upregulation of PD-L1, subsequently suppressing NK cell function and promoting T cell apoptosis [73]. As previously described, M2-polarized TAMs can transfer RNA to cancer cells by secreting exosomes. Yin et al. described an effect of long non-coding RNA nuclear paraspeckle assembly transcript 1 (NEAT1) carried by M2-TAMs-derived EVs into OC cells. After the transfer, NEAT1 could upregulate ZEB1 expression by sponging miR-101-3p. Upregulation of ZEB1 may increase the PD-L1 expression, leading to CD8^+^ T cell apoptosis and enhanced proliferative/invasive tumor behavior [74]. New findings on cisplatin resistance in ovarian cancer were reported by Sheng et al., who described a novel miRNA-145/c-Myc/PD-L1 axis. Authors noted that cisplatin-mediated down-regulation of miRNA-145 is responsible for increased PD-L1 expression via c-Myc in ovarian cancer cells, which caused T-cell apoptosis in vitro [75].

**Table 1:** miRNA in the regulation of TME immune cells. The table summarizes miRNAs’ actions regarding specific types of immune cells within the TME.

miRNA	Effect	Reference
**Macrophages**		
miRNA-125b	Intraperitoneal administration of miRNA-125b in a murine ovarian tumor model caused the repolarization of macrophages to an immune-activating phenotype	[47]
miRNA-222-3p	Activation of macrophages to M2 phenotype upon ovarian cancer-derived exosomes through SOCS3/STAT3 pathway	[48]
miRNA-223	miRNA-223 from hypoxic macrophages promoted the drug resistance of ovarian cancer cells via the PTEN-PI3K/AKT pathway	[49]
miRNA-200b	KLF6 inhibition decreased the M1 polarization, leading to an immunosuppressive response	[50]
miRNA-940	Exosomal miRNA-940 caused M2 polarization	[51]
miRNA-21-3p miRNA-125b-5p miRNA-181d-5p	Exosomes containing the mentioned miRNAs caused M2 polarization	[52]
miRNA-181c-5p	SKOV-3 derived exosomes with miRNA-181c-5p caused M2 polarization	[53]
miRNA-532-3p	CircATP2B4 served as a competing endogenous RNA of miR-532-3p and induced M2 polarization through miR-532-3p/SREBF1/PI3Ka/AKT axis	[54]
miRNA-1246	Oncogenic exosomal miRNA-1246 promoted tumor progression in the TME via M2-type macrophages	[55]
miRNA-221-3p	Exosomes from M2-type macrophages caused EOC proliferation through CDKN1B	[56]
miRNA-7	TWEAK increased miRNA-7 expression in macrophages and its’ exosomes	[59]
**T cells**		
miRNA-29a-3p miRNA-21-5p	Upon transfection of CD4^+^ T cells with equivalent miRNA mimics STAT3 suppression was observed, which influenced Treg/Th17 cell ratio	[57]
miRNA-29a-3p	Exosomal miRNA-29a-3p decreased PD-L1 through the FOXO3-AKT/GSK3β pathway	[58]
miRNA-let-7i	Overexpression of miRNA-let-71 caused the increase of T cells presence within the tumor and upregulation of APCs activity	[60]
miRNA-142	Artesunate in murine model upregulated miRNA-142, which suppressed Sirt1 and promoted Th1 differentiation	[64]
miRNA-1245	miRNA-1245 targets MCT1, reduces lactate uptake and impairs immune-suppressive responses of Tregs	[65]
miRNA-146a	miRNA-146a targets IL-1 receptor-associated kinase 1 (IRAK1) and tumor necrosis factor receptor-associated factor 6 (TRAF6) leading to NF-κB signaling inhibition; upregulation of miRNA146a leads to CD8+ T cell infiltration	[66]
**DCs**		
miRNA-22 miRNA-503	miRNA-22 targets YWHAZ and blocks PI3K/Akt and MAPK pathways miRNA-503 downregulates Bcl2 expression Such actions cause reduced survival of DCs	[61]
miRNA-155	miRNA-155 delivery to tumor-associated DCs induced inhibition of tumor progression	[62]
**NK cells**		
miRNA-20a	miRNA-20a targets MICA/B (ligands of the NKG2D receptor), which leads to NK cells’ suppression	[63]
**MDSCs**		
miRNA-211	miRNA-211 targets C/EBP homologous protein (CHOP); upregulation of miRNA-211 resulted in inhibition of MDCSs’ immunosuppressive actions	[67]

**Table 2:** miRNA in the regulation of PD-1/PD-L1 axis. The table summarizes miRNAs’ actions regarding the axis and its effect on the TME.

miRNA	Effect	Reference
**PD-1/PD-L1**		
miRNA-424(322)	miRNA-424(322) inhibited PD-L1 and CD80 expression	[68]
miRNA-576-3p	miRNA-576-3p targets PD-L1 and cyclin D1, leading to an increase in cisplatin sensitivity	[69]
miRNA-200c-3p	miRNA-200c-3- reduces PD-L1, c-Myc, and β-catenin, leading to sensitization of ovarian cancer cells to treatment	[70]
miRNA-155-5p	Low intake of miRNA-155-5p by macrophages leads to immunosuppression by upregulation of PD-L1	[71]
miRNA-34a-5p	miRNA-34a-5p negatively regulates the expression of PD-L1	[72]
miRNA-92	LATS2 as a target for miRNA-92; downregulation of LATS2 leads to an increased translocation of YAP1 and upregulation of PD-L1	[73]
miRNA-145	cisplatin-mediated down-regulation of miRNA-145 is responsible for increased PD-L1 expression via c-Myc in ovarian cancer cells	[75]

## 4. Conclusions

The TME plays a pivotal role in tumorigenesis, migration of cancer cells, tumor growth, and progression. The communication of cancer cells with infiltrating cells and stroma causes immunosuppression and tumor survival. Thus, there is still a growing need for extensive research concerning TME, driven by multilayered and complicated processes leading to cancer cell persistence or elimination. One of the most critical regulating factors within the TME are miRNAs. Aberrant expression of miRNAs in both tumor and tumor-infiltrating immune cells may cause pathological dysfunction of several signaling pathways or cancer cell-regulatory factors.

Moreover, tumor-derived exosomal miRNAs may also alter immune responses in the TME, impairing defense against cancer cells. In the review, we have highlighted the importance of miRNAs regarding the functions of immune cells in the TME. The TME also comprises endothelial cells, cancer-associated fibroblasts, and an extracellular matrix. All of those compounds take part in shaping the tumor environment. However, we highlighted the mechanisms by which miRNAs affect ovarian cancer by directly regulating immune cells within the TME. Such knowledge may not only serve as a way to identify biomarkers or elucidate pathogenesis. Still, it can also contribute to developing miRNA-based new therapeutical approaches. Special attention in preclinical studies should be given to exosome-derived miRNAs, which may be delivered to cancer cells as a new type of molecular drug. Even though multiple miRNAs influencing the ovarian cancer TME have been described so far, there is still much to be investigated. Most studies above are based on cancer cell lines or murine models. Only more detailed and extensive characterization of miRNAs in preclinical models, followed by clinical research, might be beneficial in creating new targeted therapies.
